# Molecular Packaging, Liquid-Crystalline Order, and Three-Dimensional Architecture of Dinoflagellate Chromosomes

**DOI:** 10.4014/jmb.2608.08034

**Published:** 2026-10-13

**Authors:** Jeong Min Chung, Toshinobu Suzaki, Yasuhiro Fukuda, Chihong Song

**Affiliations:** 1 Department of Biotechnology, The Catholic University of Korea, Bucheon 14662, Republic of Korea; 2 Department of Biology, Graduate School of Science, Kobe University, Kobe 657-8501, Japan; 3 Laboratory of Sustainable Animal Environment, Graduate School of Agricultural Science, Tohoku University, Osaki, Miyagi 989-6711, Japan; 4 Department of Convergence Medicine, Pusan National University School of Medicine, Yangsan 50612, Republic of Korea; 5 Core Research Facility, Pusan National University, Yangsan 50612, Republic of Korea; 6 Medical Research Institute, Pusan National University School of Medicine, Yangsan 50612, Republic of Korea

**Keywords:** Dinoflagellate, Dinokaryon, Liquid-crystalline chromosome, Histone-like protein, Volume electron microscopy, Chromosome architecture

## Abstract

Many dinoflagellates package exceptionally large nuclear genomes within the dinokaryon, a nucleus that departs from conventional eukaryotic patterns of chromosome condensation, DNA packaging, and mitosis. Their chromosomes remain visibly condensed through most or all of the cell cycle. In many core dinoflagellates, DNA exhibits cholesteric liquid-crystalline order, while canonical nucleosomal chromatin does not serve as the dominant structural framework. This review synthesizes current evidence on dinoflagellate chromosome organization, spanning molecular packaging mechanisms and three-dimensional nuclear architecture. Dinoflagellates are histone-poor rather than histone-free: divergent core histones persist at low abundance, whereas HCc/MBNP-type proteins can be highly abundant in some species, with additional contributions from dinoflagellate viral nucleoproteins, divalent cations, RNA-associated factors, and modified DNA bases. Classical electron microscopy revealed nested arch patterns consistent with cholesteric order and inspired spiral, double-helical, and toroidal chromonema models, although molecular-resolution support remains lacking and fixation-sensitive features require caution. Recent volume electron microscopy and chromosome-conformation studies are revising chromosome counts and nuclear organization. Each of four reconstructed *Oxyrrhis marina* nuclei contained 398–405 physically segmented chromosome bodies, focused ion beam scanning electron microscopy (FIB-SEM) reconstruction of a *Prorocentrum cordatum* CCMP 1329 nucleus resolved 62 tightly packed chromosome bodies together with an organized nucleolus and clustered nuclear pores, and high-throughput chromosome conformation capture (Hi-C) studies in Symbiodiniaceae revealed chromosome-scale scaffolds and transcription-dependent domains. Integrating standardized volume electron microscopy, chromosome-scale genomics, cryo-electron tomography, and biochemical reconstitution should connect molecular packaging to whole-nucleus architecture and improve understanding of ecologically important microbial eukaryotes.

## Introduction

Dinoflagellates are alveolate protists with major ecological roles in marine and freshwater ecosystems, spanning free-living phototrophs and mixotrophs, heterotrophic predators, endosymbionts, and parasites, with several lineages central to harmful algal blooms [1, 2]. Dinoflagellates also include coral symbionts whose diversity and physiological functions influence reef health and stress tolerance [3, 4]. Their nuclear cell biology is therefore directly relevant to environmental microbiology and biotechnology, including the interpretation of genome assemblies, the monitoring of bloom-forming taxa, and the development of molecular tools for ecologically important microbial eukaryotes. Understanding how they package and express their unusually large genomes has both fundamental and applied significance. The dinoflagellate nucleus, or dinokaryon, contains chromosomes that remain condensed and visible during interphase, retains its nuclear envelope through mitosis, and segregates chromosomes using extranuclear spindle microtubules that interact with the nucleus through cytoplasmic channels or envelope-associated sites [5, 6].

At the molecular level, dinoflagellate chromosomes are also atypical. In many core dinoflagellates, DNA exhibits cholesteric liquid-crystalline order, as inferred from arch-like patterns in electron micrographs and supported by birefringence and DNA-condensation measurements [7, 8]. At the same time, the nucleosome-based chromatin that organizes most eukaryotic genomes is strongly depleted. Early biochemical studies therefore described dinoflagellates as lacking histones [9], but genomic, transcriptomic and proteomic work has shown that this wording is too absolute. Core histones persist, although they are divergent and scarce, and the principal packaging burden appears to have shifted to lineage-specific or lineage-expanded basic proteins [10].

This combination poses an unusual biological challenge: some of the largest eukaryotic genomes must be replicated, transcribed and segregated while their chromosomes remain permanently condensed. Classical electron microscopy and cytochemistry produced influential models of helical or toroidal nucleofilament packing [11], but many of those observations relied on chemically fixed, resin-embedded samples. More recent work has changed the field in two ways. First, a decade of genome sequencing has revealed wide variation in genome size, gene arrangement, histone retention and chromosome-scale folding [12]. Second, whole-nucleus reconstructions now allow individual chromosomes to be counted and measured rather than inferred from two-dimensional sections [13, 14].

This review shifts the focus of dinoflagellate nuclear biology from the dinokaryon as an unusual nucleus to the architecture of the chromosomes themselves. We first discuss the molecular components that replace canonical nucleosomes as the dominant packaging system. We then evaluate models for liquid-crystalline higher-order structure, summarize emerging three-dimensional data on chromosome number and nuclear architecture, and outline how cryo-electron tomography and single-particle cryo-electron microscopy could resolve the still-unknown molecular organization of the dinoflagellate nucleofilament. This review focuses on peer-reviewed studies addressing the molecular composition, ultrastructure, three-dimensional organization, and chromosome-scale folding of dinoflagellate chromosomes, with particular emphasis on electron microscopy, volume electron microscopy, proteogenomics, and chromosome-conformation capture. Studies centered primarily on plastid genomes or population genetics are outside its scope.

Previous reviews have addressed dinokaryon biology, dinoflagellate genomics, or liquid-crystalline chromosome models largely as separate topics [5, 6, 11, 12, 15]. Here, we integrate recent volume electron microscopy reconstructions and chromosome-conformation studies with molecular packaging data to distinguish directly supported architectural features from model-dependent interpretations and to identify experimental approaches that can connect chromosome organization across structural scales.

## The Genome-Packaging Problem in the Dinokaryon

Dinoflagellate nuclear genomes span an unusually broad range. Recent genomic synthesis places most free-living dinoflagellates in the approximately 1–250 Gbp range, with smaller, more streamlined genomes in some parasitic lineages and Symbiodiniaceae [12]. Genome size alone, however, does not predict chromosome number or explain how DNA is physically accommodated within the dinokaryon. For example, *Prorocentrum cordatum* CCMP 1329 has a haploid genome assembly of approximately 4.15 Gbp with 85,849 predicted protein-coding genes, as reported in its nuclear proteogenomic study, yet focused ion beam scanning electron microscopy (FIB-SEM) reconstruction identified 62 chromosome bodies [14]. By contrast, the chromosome-scale assembly of *Symbiodinium microadriaticum* spans 624 Mb and comprises 94 high-throughput chromosome conformation capture (Hi-C)-resolved scaffolds [16].

The problem is therefore not merely that dinoflagellate genomes are large. Rather, many dinoflagellates combine large DNA content with permanent or near-permanent chromosome condensation, limited canonical nucleosomal packaging and unusual mitosis. In canonical eukaryotic chromatin, DNA is wrapped around histone octamers to form nucleosomes, and higher-order folding of nucleosome fibers is dynamic and does not require a regular 30-nm fiber in vivo [17]. Dinoflagellates approach the problem differently. They retain histone genes but use a nucleosome-depleted architecture in which DNA organization likely involves basic proteins, divalent cations, RNA-associated components and liquid-crystalline packing [10, 14].

Genome-expansion mechanisms also appear diverse. Dinoflagellate genome-size evolution has been linked to repetitive-DNA accumulation and genome duplication rather than to a single universal mechanism [12, 18]. This diversity cautions against using any one species as a general proxy for the dinokaryon and highlights the need to distinguish general principles of chromosome packaging from lineage- or species-specific solutions.

## Molecular Components of Dinoflagellate Chromosome Compaction

### Residual Histones and a Nucleosome-Depleted Chromatin State

Dinoflagellate chromatin is best described as nucleosome-depleted and histone-poor, rather than histone-null. Core histone genes are present and transcribed in multiple dinoflagellate lineages, and their histone-related machinery includes divergent variants and chromatin-modifying enzymes [19, 20]. In *P. cordatum*, the genome encodes all four core histones (H2A, H2B, H3 and H4), and proteomic analysis of enriched nuclei detected H2A, H2B and H4, although at very low abundance [14]. These observations demonstrate that histones persist at low abundance in dinoflagellates, although their specific functions remain unresolved. Transcriptomic studies have identified all four core histones together with histone-modifying and nucleosome-associated proteins, leading to the proposal that residual histone-based machinery may contribute to gene-expression regulation rather than bulk genome packaging [21]. In addition, an expressed sequence tag study identified rare transcripts encoding an H2A.X-like protein in *Alexandrium tamarense* and proposed, based on the conserved features and known functions of H2A.X in other eukaryotes, that this histone variant may participate in DNA double-strand break repair [22]. Importantly, these proposed regulatory and DNA-repair functions have not yet been experimentally demonstrated in dinoflagellates and should therefore be regarded as functional hypotheses rather than established roles.

The distinction is important. Earlier statements that dinoflagellates lack histones altogether are historically understandable, given the extreme depletion of canonical nucleosomes, but they are no longer accurate [23]. Conversely, the mere presence of histone genes should not be taken as evidence for conventional canonical chromatin. The key biological feature is therefore a shift in the dominant mode of DNA condensation away from canonical nucleosomes toward noncanonical DNA-binding proteins. Among these, HCc/MBNP-type proteins are thought to contribute substantially to bulk chromosome compaction in several studied taxa, whereas DVNPs may have contributed to the evolutionary transition away from conventional nucleosome-based packaging. Divalent cations, RNA-associated components, and liquid-crystalline higher-order organization may provide additional contributions to chromosome condensation [10, 14].

### Major Basic Nuclear Proteins, Histone-Like Proteins, and HCc/MBNP-Type Proteins

The most abundant candidate packaging proteins in several studied dinoflagellates are major basic nuclear proteins (MBNPs), also called histone-like proteins or HCc-type proteins after their characterization in *Crypthecodinium cohnii* [24]. These proteins are not canonical histones; they differ in sequence, biochemical properties and evolutionary origin, but they bind DNA and can promote concentration-dependent DNA compaction *in vitro* [25].

The *P. cordatum* nuclear proteome provides one of the clearest quantitative examples. Among proteins assigned to nuclear functions, HCc2-like major basic nuclear proteins were the most abundant; the genome contains eleven genes of this class, seven were detected proteomically, and these proteins were approximately 29-fold enriched in nuclear fractions relative to whole-cell fractions [14]. This abundance identifies HCc/MBNP-type proteins as leading candidates to carry much of the structural load in at least some core dinoflagellates. At the same time, their exact mode of DNA binding in situ, their stoichiometry on the nucleofilament, and their relationship to liquid-crystalline ordering remain unresolved.

### Dinoflagellate Viral Nucleoproteins

Dinoflagellate viral nucleoproteins (DVNPs) are small, highly basic proteins of apparent viral origin whose emergence has been proposed to coincide with the evolutionary reduction of nucleosome-based condensation [10]. Functional work in heterologous systems showed that a representative DVNP can bind chromatin, localize to histone-associated sites, promote nucleosome displacement, and perturb transcription, making it a plausible contributor to histone depletion during dinoflagellate evolution [26]. However, this does not mean that DVNPs are the dominant structural proteins in every dinoflagellate nucleus. In *P. cordatum*, the genome encodes eleven DVNP homologs, but only one was detected in the nuclear proteome and at low abundance, arguing against a major bulk-compaction role in that species [14].

A balanced model must therefore account for species-dependent differences in nuclear protein composition. DVNPs may have contributed to the evolutionary transition away from conventional nucleosomes, whereas present-day chromosome architecture may rely more strongly on other lineage-expanded basic proteins, particularly HCc/MBNP-type proteins, in some taxa.

### Divalent Cations, RNA-Associated Factors, Modified Bases, and Additional Chromatin-Associated Factors

Nonprotein factors also contribute to chromosome stability. Classic biochemical work showed that Ca^2+^ and Mg^2+^ help stabilize native dinoflagellate chromosome structure [27]. High-resolution ion-probe mass spectrometry mapped divalent cations within dinoflagellate chromosomes, supporting the idea that the ionic environment contributes directly to chromosome compaction [28]. Proteomic analyses of dinoflagellate chromatin also revealed abundant RNA-binding proteins, consistent with a strong connection between RNA metabolism and chromosome architecture [29]. Dinoflagellate nuclear DNA also contains unusual modified bases, and both B- and Z-form DNA have been reported within chromosomes; these features may influence local packing and the partition between structural and transcriptionally engaged DNA [12, 30]. Recent genome-wide mapping in *B. minutum* showed that 5-hydroxymethyluracil (5-hmU) is enriched over selected repetitive elements and often occurs near gene-array and dinoflagellate topologically associating domain (dinoTAD) boundaries, while elevated 5-hmU levels are generally associated with reduced chromatin accessibility. These findings link a characteristic dinoflagellate DNA modification to local chromosome organization, although its causal role remains unresolved [31].

At the ultrastructural level, isolated dinoflagellate chromatin does not show the bead-on-a-string pattern of canonical nucleosomal fibers. Instead, isolated dinoflagellate chromatin has been described as a relatively homogeneous nucleofilament only slightly thicker than naked DNA, on the order of a few nanometers [32]. In freeze-substituted *O. marina*, nucleofilaments associated with chromosome–nucleolus contacts were measured at approximately 6.5 nm [13]. How this nucleofilament is built at the molecular level remains a central unresolved question. A working hypothesis is that DNA is coated by basic proteins and stabilized by cations and RNA-associated factors, but the precise architecture of this nucleofilament and the way it accommodates ongoing transcription and replication within persistently condensed chromosomes require direct structural determination [33]. Earlier cytological and molecular studies of dinoflagellate genome organization and gene expression suggested a structural and functional differentiation within dinoflagellate chromosomes, with transcriptionally engaged DNA proposed to occupy locally accessible regions while the chromosomes remain globally condensed [33]. This provides a possible framework by which gene expression can occur within an otherwise highly compacted chromosome. In contrast, the structural changes that permit DNA replication within persistently condensed dinoflagellate chromosomes remain less well characterized. Direct visualization of replication-associated local chromosome remodeling will therefore be important for connecting chromosome architecture with genome function.

Together, these observations indicate that dinoflagellate chromosome compaction is not driven by a single replacement for histones, but by a combination of residual histones, lineage-expanded basic proteins, DVNPs, divalent cations, RNA-associated factors and unusual DNA features. These molecular components and their proposed functional implications are summarized in Table 1.

## Liquid-Crystalline Higher-Order Architecture

The most distinctive mesoscale feature of core dinoflagellate chromosomes is their liquid-crystalline organization. Thin sections often show nested, arch-shaped bands, interpreted as sectioning views through a cholesteric arrangement in which locally parallel nucleofilaments rotate in orientation between layers [7]. Birefringence and DNA-condensation measurements support the view that both macroscopic chromosome organization and ultrastructural DNA packing contribute to the optical behavior of these chromosomes [8].

Several structural models have attempted to connect the visible arch pattern with an underlying nucleofilament path. Dodge proposed a spiral chromonema model based on observations of dinophycean chromosomes [34]. Later observations of spread chromosomes were interpreted as supporting double-helical arrangements [35], and Oakley and Dodge proposed a double-helically coiled toroidal chromonema [36]. Wong integrated these ideas into a liquid-crystalline chromosome model comprising a birefringent inner core, a core-surface compartment containing a spiraling chromonema, and a peripheral compartment carrying gene-containing loops [15]. These models are valuable because they provide testable geometries, but they should be presented as models rather than as solved molecular structures.

Two limitations are especially important for a modern review. First, most classic images were obtained from chemically fixed and sectioned material. Second, the observed arch pattern is not universal across dinoflagellate lineages. This contrast is illustrated by the periodic arch-shaped chromosome filaments in the core dinoflagellate *Heterocapsa circularisquama* and the less regularly organized chromosome material in the early-diverging *Oxyrrhis marina* (Fig. 1A and B). Consistent with this comparison, freeze-substituted samples of *O. marina* did not show the repeating arch-shaped liquid-crystalline pattern observed in many core dinoflagellates [13].

Together, these observations indicate that dinoflagellate chromosome architecture is heterogeneous across lineages. The absence of a regular arch-shaped pattern in *O. marina* may reflect a lineage-specific or early-diverging condition, but *O. marina* is not a direct ancestor of core dinoflagellates. Broader taxonomic sampling using comparable preservation and imaging methods will therefore be required before the evolutionary sequence of chromosome architectural transitions can be inferred [13, 37, 38].

## Chromosome Number and Nuclear Architecture from Three-Dimensional Methods

Chromosome number has been one of the most difficult parameters to define consistently in dinoflagellates because many species contain numerous small chromosomes that remain persistently condensed and are difficult to resolve by light microscopy. In addition, chromosomes are densely packed within the nucleus and often overlap in two-dimensional preparations, making it difficult to distinguish individual chromosome bodies in thin sections, chromosome spreads, 4′,6-diamidino-2-phenylindole (DAPI)-stained nuclei or fluorescence in situ hybridization (FISH) images. Reported values can also vary with life-cycle stage, growth stage, ploidy, culture condition and nuclear morphology, because chromosome condensation, nuclear organization and chromosome separability may change depending on the biological state of the cell. Preparation-related factors, including fixation, sectioning artifacts, imaging modality and counting criteria, further complicate direct comparison among studies [13, 14, 16, 39]. Recent studies have therefore moved toward whole-nucleus three-dimensional reconstruction and complementary genome-scale approaches. The following sections review this transition through representative examples: freeze-substitution and volume EM in *Oxyrrhis marina*, FIB-SEM reconstruction and nuclear proteogenomics in *Prorocentrum cordatum*, chromosome-conformation data in Symbiodiniaceae, and nuclear landmarks such as telomeres, nucleoli and the nuclear envelope. Representative physical chromosome counts, chromosome-scale assemblies, and structural measurements from these studies are summarized in Table 2.

### *Oxyrrhis marina*: a Major Revision by Freeze-substitution and Volume EM

*Oxyrrhis marina* is widely used as an informative representative of an early-diverging dinoflagellate lineage for studies of dinokaryon evolution [37, 38]. Classical chromosome counting reported approximately 55 chromosomes [40]. Freeze-substitution combined with volume electron microscopy revised this value dramatically. In four reconstructed cells, volume EM identified 398, 405, 402, and 403 physically segmented chromosome bodies, corresponding to a range of 398–405 (Fig. 1C) [13].

The same study showed why earlier values were likely underestimated. Many *O. marina* chromosomes are short and thin, approximately 0.1 μm wide and 0.1–2.5 μm long, with a median length of 0.73 μm, and roughly 80% fall between 0.2 and 1.4 μm [13]. Conventional chemical fixation produced electron-dense granules previously interpreted as fragmented chromosomes, but these granules were absent after freeze-substitution, indicating that some apparent fragments were preparation artifacts and that short chromosomes had probably been overlooked or under-resolved in earlier counts [13]. The *O. marina* nucleus also contains a central nucleolus to which multiple chromosomes attach, with nucleofilaments observed penetrating the nucleolus (Fig. 1D) [13]. Methodologically, this work illustrates that reliable chromosome counting in dinoflagellates benefits from preservation methods that minimize fixation artifacts, whole-nucleus three-dimensional reconstruction, and segmentation approaches capable of resolving numerous small chromosomes within a densely packed dinokaryon.

### *Prorocentrum cordatum*: FIB-SEM Reconstruction and Nuclear Proteogenomics

In the core dinoflagellate *P. cordatum* CCMP 1329, high-resolution FIB-SEM analysis reconstructed a flattened nucleus containing 62 tightly packed chromosome bodies with individual volumes of approximately 0.4–6.7 μm^3^ and a mean volume of 2.7 μm^3^. Together, the chromosomes occupied about 80% of the nuclear interior, which had a volume of approximately 217 μm^3^ (Fig. 1E) [14]. Several chromosomes were spatially associated with the nucleolus (Fig. 1F). The reconstruction also revealed other nuclear structures, including a conspicuous feature interpreted cautiously as a possible extension of the endoplasmic reticulum into the nucleus [14].

The study also mapped the nuclear envelope. Nuclear pore complexes were not randomly distributed but concentrated in patches near the nucleolus, with 475 pores counted across the envelope [14]. The spatial proximity of nuclear pores, the nucleolus, and selected chromosomes raises the possibility that chromosome positioning is coordinated with nucleocytoplasmic transport and ribosome biogenesis, although direct functional evidence is still lacking. Because *P. cordatum* also has a proteogenomic dataset from enriched nuclei, it provides a useful bridge between whole-nucleus morphology and candidate nuclear proteins. This pairing of three-dimensional reconstruction with proteomics distinguishes the study from earlier, primarily morphological studies [14].

### Chromosome-Conformation Data in Symbiodiniaceae

Chromosome-conformation capture provides a genomic dimension to electron microscopy. In *S. microadriaticum*, Hi-C grouped, ordered, and oriented sequence scaffolds into 94 chromosome-scale scaffolds, close to earlier electron-microscopy-based chromosome estimates of 97 ± 2 [16]. Genes were enriched toward chromosome ends and arranged in alternating unidirectional blocks, linking gene organization to chromosome architecture [16]. In *B. minutum*, Hi-C scaffolding identified 91 major pseudochromosomes of at least 500 kb, encompassing approximately 94% of the total assembled sequence; these represent genome-assembly units rather than chromosome bodies counted microscopically [39]. Hi-C further revealed large dinoflagellate topologically associating domains (dinoTADs) without conventional chromatin loops. These domains are associated with convergent gene-array boundaries, providing a direct connection between transcriptional organization and chromosome-scale topology. Importantly, transcriptional inhibition disrupted dinoTAD organization, supporting transcription-induced supercoiling as a major force contributing to chromosome folding [39]. Thus, persistent chromosome condensation does not imply structural inactivity. Instead, transcription can itself contribute to higher-order chromosome organization through local and domain-scale topological changes. How comparable structural remodeling accommodates DNA replication remains much less well understood and represents an important area for future investigation.

Together, these studies show that dinoflagellate chromosomes cannot be understood only as static condensed bodies. They also possess chromosome-scale domains whose organization is linked to transcriptional activity. However, a physically segmented chromosome body, a chromosome-scale scaffold, and a pseudochromosome are not interchangeable measurements. Integrating Hi-C with volume electron microscopy and, ultimately, cryo-electron tomography will be essential for connecting transcription-linked chromosome topology with chromosome territories, nucleolar contacts, and liquid-crystalline nucleofilament architecture.

### Nuclear Landmarks for Chromosome Positioning: Telomeres, Nucleoli and the Nuclear Envelope

Telomere and nucleolar markers provide additional landmarks. FISH studies localized plant-type telomeric repeats on dinoflagellate chromosomes and suggested structural implications for chromosome ends [41]. Broader analyses of dinoflagellate genome spatial organization place these chromosome ends within the overall nuclear architecture [42]. Telomere and telomerase analyses confirmed maintenance of these repeats in liquid-crystalline chromosomes [43]. Recent work on dinochromosome heterotermini and telosomal anchorage suggests that one chromosome end is positioned at or within the nuclear envelope during dinomitosis and that telomeric regions may constitute the principal nucleosomal domains [44]. These observations fit older cytological descriptions of closed mitosis with an extranuclear spindle [5], although the molecular components of the telomere-envelope linkage remain poorly defined.

The nucleolus is another recurring reference point. As described above, multiple chromosomes attach to the central nucleolus in *O. marina*, whereas several chromosomes are spatially associated with the nucleolus in *P. cordatum* [13, 14]. Earlier work on *Prorocentrum micans* localized rRNA coding sequences in the nucleolus and described nucleolar behavior through the cell cycle [45, 46]. In the genus *Karenia*, flow cytometry and FISH/non-denaturing fluorescence in situ hybridization (ND-FISH) mapping of telomeric, 45S ribosomal DNA (rDNA) and microsatellite markers proved informative for chromosome behavior even though clean chromosome counts were difficult because the nuclear envelope persists [47]. Together these studies suggest that ribosomal-DNA-bearing chromosome regions and nucleolar attachment are important organizing features, but the extent to which nucleolar contacts define chromosome territories across species remains unknown.

## Technical Priorities for the Next Phase

The field now needs methods that connect scales while minimizing preparation- and interpretation-related artifacts. Volume electron microscopy can count whole chromosomes and map nuclear topology, but resin embedding, staining, and segmentation can affect fine-structural interpretation. Freeze-substitution improves preservation relative to conventional chemical fixation in some contexts, as shown clearly for *O. marina* [13], but it still does not provide the native hydrated state. Cryo-focused ion beam milling followed by cryo-electron tomography could image vitrified nuclei or chromosomes without chemical fixation and test whether local nucleofilament organization shows features consistent with the helical or toroidal geometries proposed from classical sections [15, 35, 36].

For defined molecular components, biochemical reconstitution could provide a complementary route, with single-particle cryo-electron microscopy becoming useful if stable protein-DNA complexes can be obtained. Purified HCc/MBNP-type proteins, DVNPs and defined DNA substrates could reveal how individual proteins coat, bend, bridge or bundle DNA. Such structures would need to be interpreted alongside in situ cryo-electron tomography, because native chromosomes also contain divalent cations and RNA-associated proteins [27–29], as well as higher-order liquid-crystalline constraints [7, 15]. Because these approaches interrogate dinoflagellate chromosome organization at different and complementary structural scales, their principal contributions, strengths, and limitations are summarized in Table 3.

Finally, the reporting of chromosome counts should be standardized. For each species, future reports should state the growth stage, life-cycle stage, fixation or vitrification strategy, imaging modality, segmentation criteria, and whether a reported value represents physical chromosome bodies, FISH-defined telomere pairs, or chromosome-scale genomic scaffolds. Without this information, chromosome numbers from different studies cannot be compared directly [13, 16]. No single method spans the full range from molecular packaging to whole-nucleus organization; therefore, integrating molecular, genomic, and three-dimensional imaging approaches will be essential for establishing a multiscale structural model of the dinoflagellate chromosome.

## Concluding Remarks

Dinoflagellate chromosomes represent diverse noncanonical eukaryotic solutions to genome packaging. Across the group, persistent condensation and histone-poor, nucleosome-depleted organization are recurrent features, whereas regular cholesteric liquid-crystalline order is best supported in many core dinoflagellates. Their nuclei also show nonrandom spatial relationships among chromosomes, nucleoli, nuclear pores, and chromosome ends, and many species possess exceptionally large genomes. Four conclusions emerge from the available evidence: dinoflagellates are histone-poor rather than histone-free; the abundance and probable structural roles of DVNPs and HCc/MBNP-type proteins vary among species and remain incompletely resolved; cholesteric liquid-crystalline order is supported at the mesoscale in many core dinoflagellates, whereas specific helical or toroidal nucleofilament paths remain unverified molecular models; and reported chromosome counts depend strongly on the method used. Recent volume electron microscopy and chromosome-conformation studies provide a stronger basis for making these distinctions. The next major advance will likely come from integrating standardized whole-nucleus reconstructions, cryo-electron tomography, and structural analyses of the proteins that coat and organize dinoflagellate DNA. Connecting these scales should also improve interpretation of chromosome-scale assemblies and molecular responses in bloom-forming and symbiotic dinoflagellates.

## Figures and Tables

**Fig. 1:**
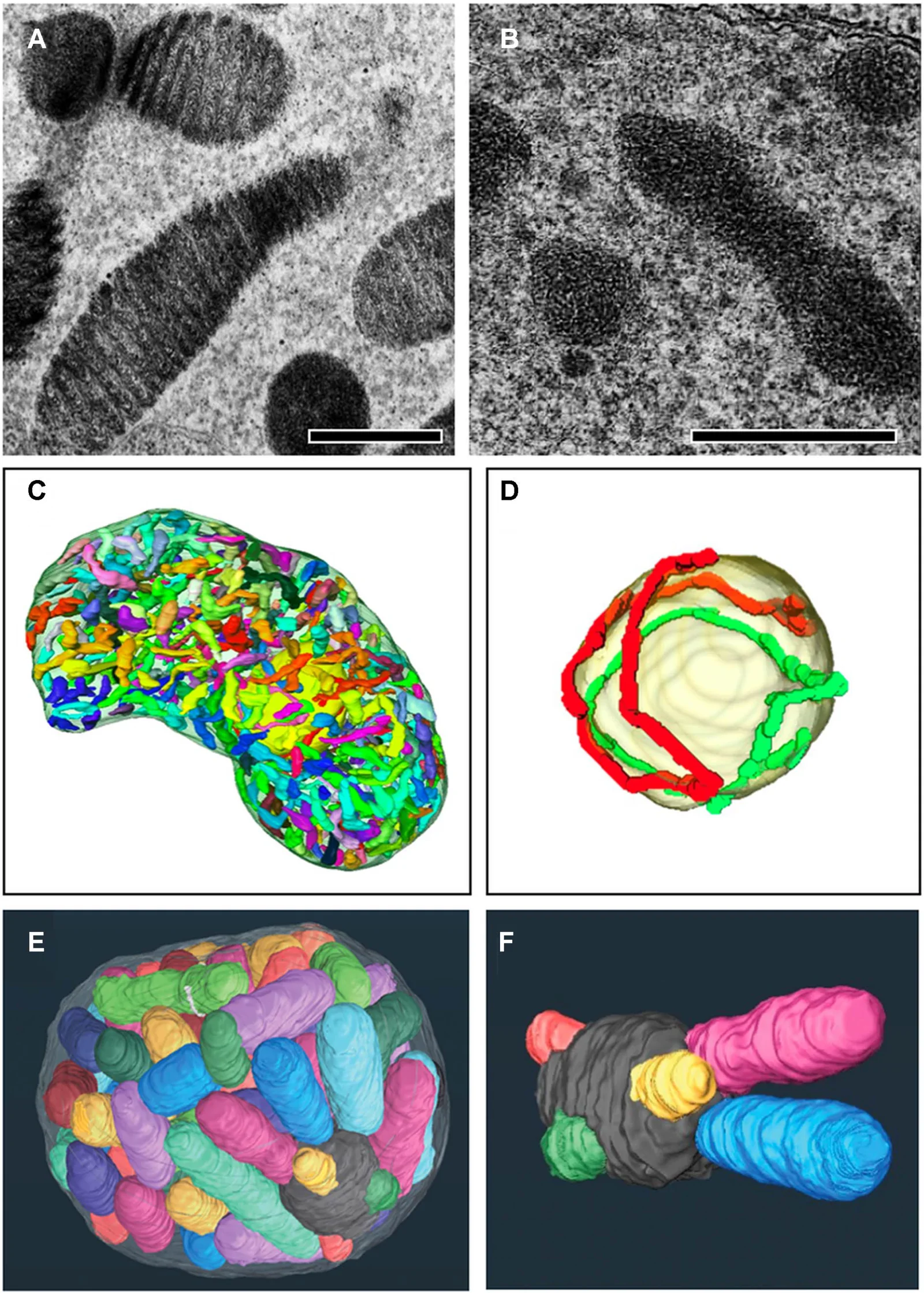
Ultrastructural diversity and three-dimensional nuclear architecture of dinoflagellate chromosomes. (**A, B**) Comparison of chromosome ultrastructure between a core dinoflagellate and an early-diverging dinoflagellate prepared by conventional chemical fixation. (**A**) Chromosome morphology of the core dinoflagellate *Heterocapsa circularisquama*, showing periodic arch-shaped filaments characteristic of many dinokaryon chromosomes. (**B**) Chromosome morphology of the early-diverging dinoflagellate *Oxyrrhis marina*, showing filamentous chromosome material without a regular periodic pattern. Scale bars: 500 nm in (**A, B**). (**C, D**) Three-dimensional reconstruction of the nucleus and nucleolus of *O. marina* based on serial-section volume electron microscopy. (**C**) Whole-nucleus reconstruction showing numerous chromosomes distributed throughout the nucleus; individual chromosomes are shown in different colors. (**D**) Three-dimensional reconstruction of the nucleolus with associated chromosomes, illustrating chromosome-nucleolus contacts in *O. marina*. (**E, F**) Three-dimensional reconstruction of the nucleus of *Prorocentrum cordatum* based on FIB-SEM imaging. (**E**) Whole-nucleus reconstruction showing tightly packed chromosomes within the dinokaryon. Individual chromosomes are arbitrarily colored, the nucleolus is shown in dark gray, and the nuclear envelope is displayed transparently. (**F**) Enlarged view of the nucleolus and selected nucleolus-associated chromosomes, highlighting their spatial proximity. Panels (**A-D**) were adapted from Fukuda *et al*. [13] under the Creative Commons Attribution 4.0 International License (CC BY 4.0). Panels (**E, F**) were adapted from Kalvelage *et al*. [14] under the CC BY 4.0 license.

**Table 1:** Key molecular features of dinoflagellate chromosome compaction.

Feature	Key evidence	Functional implication
Residual histones	Core histone genes are present; H2A, H2B, and H4 occur at low abundance in *P. cordatum* nuclei [14, 19, 20].	Potential localized roles in transcription-associated chromatin and/or DNA repair; direct functional evidence in dinoflagellates remains limited [21, 22]
HCc/MBNP-type proteins	Abundant basic nuclear proteins bind and compact DNA in vitro [14, 24, 25].	Leading candidates for structural compaction
DVNPs	A representative DVNP binds chromatin and can promote nucleosome displacement in heterologous systems [10, 26].	Possible contribution to evolutionary nucleosome reduction
Divalent cations	Ca^2+^ and Mg^2+^ stabilize native chromosomes [27, 28].	Ionic stabilization of compacted DNA
RNA-associated factors	RNA-binding proteins are enriched in chromatin [29].	Possible coupling of RNA metabolism and structure
Modified bases and B-/Z-DNA	Modified bases, including 5-hmU, and alternative DNA conformations are reported [12, 30, 31].	Possible effects on packing and accessibility
Nucleofilament	Non-nucleosomal filament; ~6.5 nm at chromosome–nucleolus contacts in *O. marina* [13].	Candidate basic structural unit

Abbreviations: DVNP, dinoflagellate viral nucleoprotein; HCc, histone-like protein HCc; MBNP, major basic nuclear protein.

**Table 2:** Representative chromosome counts, chromosome-scale assemblies, and structural landmarks in dinoflagellates.

Taxon	Genome / assembly	Physical count or genomic assembly	Key method and structural findings
*Oxyrrhis marina* (early-diverging)	Genome size not reported in the cited volume-EM study.	398–405 chromosome bodies in four reconstructed cells [13].	Freeze-substitution serial TEM/SBF-SEM; ~0.1 μm wide and 0.1–2.5 μm long (median 0.73 μm); no regular arch pattern [13].
*Prorocentrum cordatum* CCMP 1329 (core)	Haploid assembly ~4.15 Gbp; 85,849 predicted protein-coding genes [14].	62 physically segmented chromosome bodies [14].	FIB-SEM; volumes 0.4–6.7 μm^3^ (mean 2.7 μm); chromosomes occupied ~80% of the 217 μm^3^ nuclear interior; 475 pores clustered near the nucleolus [14].
*Symbiodinium microadriaticum* (core; coral symbiont)	Chromosome-scale assembly spanning 624 Mb [16].	94 chromosome-scale scaffolds, close to an earlier EM estimate of 97 ± 2 [16].	Genes are enriched near chromosome ends and arranged in alternating unidirectional blocks, with rod-like folding and transcription-associated domains [16].
*Breviolum minutum* (core; coral symbiont)	Chromosome-scale organization analyzed by Hi-C [39].	91 major pseudochromosomes (≥500 kb), encompassing ~94% of the assembled sequence [39].	Assembly units rather than microscopy counts; Hi-C revealed dinoTADs without canonical loops, and transcriptional inhibition disrupted dinoTADs [39].

**Table 3:** Complementary approaches for investigating dinoflagellate chromosome architecture across structural scales.

Approach	Information obtained	Major strength	Major limitation
Nuclear proteomics / proteogenomics [14, 29]	Nuclear protein composition and relative enrichment	Identifies candidate chromosome-packaging proteins	Limited spatial and direct functional information
Volume EM (serial TEM/SBF-SEM/FIB-SEM) [13, 14]	3D chromosome number, size, and nuclear organization	Direct whole-nucleus visualization and segmentation	Preparation and segmentation can affect interpretation
Hi-C / chromosome-scale genomics [16, 39]	Chromosome-scale scaffolds and transcription-linked topology	Links genome organization with 3D chromosome folding	Does not directly visualize chromosome ultrastructure
Cryo-FIB / cryo-electron tomography	Near-native nucleofilament and local chromosome ultrastructure	Molecular-scale imaging without chemical fixation	Technically demanding and limited in volume
Biochemical reconstitution / single-particle cryo-EM	Protein-DNA interactions [25] and defined complex structures	Can resolve molecular mechanisms at high resolution	May not reproduce the native chromosome environment

Abbreviations: 3D, three-dimensional; EM, electron microscopy; TEM, transmission electron microscopy; SBF-SEM, serial block-face scanning electron microscopy; FIB-SEM, focused ion beam scanning electron microscopy; Hi-C, high-throughput chromosome conformation capture; cryo-FIB, cryogenic focused ion beam; cryo-EM, cryo-electron microscopy.

## References

[1] Burki F, Roger AJ, Brown MW, Simpson AGB (2020). The new tree of eukaryotes. Trends Ecol. Evol..

[2] Taylor FJR, Hoppenrath M, Saldarriaga JF (2008). Dinoflagellate diversity and distribution. Biodivers. Conserv..

[3] Stat M, Morris E, Gates RD (2008). Functional diversity in coral-dinoflagellate symbiosis. Proc. Natl. Acad. Sci. USA.

[4] LaJeunesse TC, Parkinson JE, Gabrielson PW, Jeong HJ, Reimer JD, Voolstra CR (2018). Systematic revision of Symbiodiniaceae highlights the antiquity and diversity of coral endosymbionts. Curr. Biol..

[5] Fukuda Y, Suzaki T. 2015. Unusual features of dinokaryon, the enigmatic nucleus of dinoflagellates, pp. 23-45. In Ohtsuka S, Suzaki T, Horiguchi T, Suzuki N, Not F (eds.). Marine Protists: Diversity and Dynamics. Springer Japan, Tokyo, Japan. https://doi.org/10.1007/978-4-431-55130-0_2. 10.1007/978-4-431-55130-0_2

[6] Soyer-Gobillard MO (2025). Some insights into the inventiveness of dinoflagellates: Coming back to the cell biology of these protists. Microorganisms.

[7] Bouligand Y, Soyer MO, Puiseux-Dao S (1968). La structure fibrillaire et l'orientation des chromosomes chez les Dinoflagelles. Chromosoma.

[8] Chow MH, Yan KTH, Bennett MJ, Wong JTY (2010). Birefringence and DNA condensation of liquid crystalline chromosomes. Eukaryot. Cell.

[9] Rizzo PJ, Nooden LD (1974). Partial characterization of dinoflagellate chromosomal proteins. Biochim. Biophys. Acta.

[10] Gornik SG, Ford KL, Mulhern TD, Bacic A, McFadden GI, Waller RF (2012). Loss of nucleosomal DNA condensation coincides with appearance of a novel nuclear protein in dinoflagellates. Curr. Biol..

[11] Costas E, Goyanes VJ (2005). Architecture and evolution of dinoflagellate chromosomes: an enigmatic origin. Cytogenet. Genome Res..

[12] Lin S (2024). A decade of dinoflagellate genomics illuminating an enigmatic eukaryote cell. BMC Genomics.

[13] Fukuda Y, Suzaki T, Murata K, Song C (2025). Novel ultrastructural features of the nucleus of the ancestral dinoflagellate *Oxyrrhis marina* as revealed by freeze substitution fixation and volume electron microscopy. Front. Protistol..

[14] Kalvelage J, Woehlbrand L, Schoon RA, Zink FM, Correll C, Senkler J, *et al*. 2023. The enigmatic nucleus of the marine dinoflagellate *Prorocentrum cordatum*. *mSphere* **8:** e00038-23. https://doi.org/10.1128/msphere.00038-23. 10.1128/msphere.00038-23 37358287 PMC10449503

[15] Wong JTY (2019). Architectural organization of dinoflagellate liquid crystalline chromosomes. Microorganisms.

[16] Nand A, Zhan Y, Salazar OR, Aranda M, Voolstra CR, Dekker J (2021). Genetic and spatial organization of the unusual chromosomes of the dinoflagellate *Symbiodinium microadriaticum*. Nat. Genet..

[17] Maeshima K, Ide S, Babokhov M (2019). Dynamic chromatin organization without the 30-nm fiber. Curr. Opin. Cell Biol..

[18] Hou Y, Ji N, Zhang H, Shi X, Han H, Lin S (2019). Genome size-dependent PCNA gene copy number in dinoflagellates and molecular evidence of retroposition as a major evolutionary mechanism. J. Phycol..

[19] Roy S, Morse D (2013). Transcription and maturation of mRNA in dinoflagellates. Microorganisms.

[20] Marinov GK, Lynch M (2016). Diversity and divergence of dinoflagellate histone proteins. G3 (Bethesda).

[21] Lin S, Zhang H, Zhuang Y, Tran B, Gill J (2010). Spliced leader-based metatranscriptomic analyses lead to recognition of hidden genomic features in dinoflagellates. Proc. Natl. Acad. Sci. USA.

[22] Hackett JD, Scheetz TE, Yoon HS, Soares MB, Bonaldo MF, Casavant TL (2005). Insights into a dinoflagellate genome through expressed sequence tag analysis. BMC Genomics.

[23] Rizzo PJ (2003). Those amazing dinoflagellate chromosomes. Cell Res..

[24] Sala-Rovira M, Geraud ML, Caput D, Jacques F, Soyer-Gobillard MO, Vernet G (1991). Molecular cloning and immunolocalization of two variants of the major basic nuclear protein (HCc) from the histone-less eukaryote *Crypthecodinium cohnii* (Pyrrhophyta). Chromosoma.

[25] Chan YH, Wong JTY (2007). Concentration-dependent organization of DNA by the dinoflagellate histone-like protein HCc3. Nucleic Acids Res..

[26] Irwin NAT, Martin BJE, Young BP, Browne MJG, Flaus A, Loewen CJR (2018). Viral proteins as a potential driver of histone depletion in dinoflagellates. Nat. Commun..

[27] Herzog M, Soyer MO (1983). The native structure of dinoflagellate chromosomes and their stabilization by Ca^2+^ and Mg^2+^ cations. Eur. J. Cell Biol..

[28] Levi-Setti R, Gavrilov KL, Rizzo PJ (2008). Divalent cation distribution in dinoflagellate chromosomes imaged by high-resolution ion probe mass spectrometry. Eur. J. Cell Biol..

[29] Beauchemin M, Morse D (2018). A proteomic portrait of dinoflagellate chromatin reveals abundant RNA-binding proteins. Chromosoma.

[30] Soyer-Gobillard MO, Geraud ML, Coulaud D, Barray M, Theveny B, Revet B (1990). Location of B- and Z-DNA in the chromosomes of a primitive eukaryote dinoflagellate. J. Cell Biol..

[31] Marinov GK, Chen X, Swaffer MP, Xiang T, Grossman AR, Greenleaf WJ (2024). Genome-wide distribution of 5-hydroxymethyluracil and chromatin accessibility in the *Breviolum minutum* genome. Genome Biol..

[32] Rizzo PJ, Burghardt RC (1980). Chromatin structure in the unicellular algae *Olisthodiscus luteus*, *Crypthecodinium cohnii* and *Peridinium balticum*. Chromosoma.

[33] Moreno Diaz de la Espina S, Alverca E, Cuadrado A, Franca S (2005). Organization of the genome and gene expression in a nuclear environment lacking histones and nucleosomes: the amazing dinoflagellates. Eur. J. Cell Biol..

[34] Dodge JD (1963). Chromosome structure in the Dinophyceae. I. The spiral chromonema. Arch. Mikrobiol..

[35] Livolant F, Bouligand Y (1980). Double helical arrangement of spread dinoflagellate chromosomes. Chromosoma.

[36] Oakley BR, Dodge JD (1979). Evidence for a double-helically coiled toroidal chromonema in the dinoflagellate chromosome. Chromosoma.

[37] Saldarriaga JF, McEwan ML, Fast NM, Taylor FJR, Keeling PJ (2003). Multiple protein phylogenies show that *Oxyrrhis marina* and *Perkinsus marinus* are early branches of the dinoflagellate lineage. Int. J. Syst. Evol. Microbiol..

[38] Bachvaroff TR, Gornik SG, Concepcion GT, Waller RF, Mendez GS, Lippmeier JC (2014). Dinoflagellate phylogeny revisited: using ribosomal proteins to resolve deep branching dinoflagellate clades. Mol. Phylogenet. Evol..

[39] Marinov GK, Trevino AE, Xiang T, Kundaje A, Grossman AR, Greenleaf WJ (2021). Transcription-dependent domain-scale three-dimensional genome organization in the dinoflagellate *Breviolum minutum*. Nat. Genet..

[40] Dodge JD (1963). Chromosome numbers in some marine dinoflagellates. Bot. Mar..

[41] Alverca E, Cuadrado A, Jouve N, Franca S, Moreno Diaz de la Espina S (2007). Telomeric DNA localization on dinoflagellate chromosomes: structural and evolutionary implications. Cytogenet. Genome Res..

[42] Lin S, Song B, Morse D (2021). Spatial organization of dinoflagellate genomes: novel insights and remaining critical questions. J. Phycol..

[43] Fojtova M, Wong JTY, Dvorackova M, Yan KTH, Sykorova E, Fajkus J (2010). Telomere maintenance in liquid crystalline chromosomes of dinoflagellates. Chromosoma.

[44] Kwok ACM, Yan KTH, Wen S, Sun S, Li C, Wong JTY (2024). Dinochromosome heterotermini with telosomal anchorages. Int. J. Mol. Sci..

[45] Geraud ML, Herzog M, Soyer-Gobillard MO (1991). Nucleolar localization of rRNA coding sequences in *Prorocentrum micans* Ehr. (dinomastigote, kingdom Protoctist) by in situ hybridization. Biosystems.

[46] Soyer-Gobillard MO, Geraud ML (1992). Nucleolus behaviour during the cell cycle of a primitive dinoflagellate eukaryote, *Prorocentrum micans* Ehr., seen by light microscopy and electron microscopy. J. Cell Sci..

[47] Cuadrado A, De Bustos A, Figueroa RI (2019). Chromosomal markers in the genus *Karenia*: towards an understanding of the evolution of the chromosomes, life cycle patterns and phylogenetic relationships in dinoflagellates. Sci. Rep..

